# Theranostics Based on Magnetic Nanoparticles and Polymers: Intelligent Design for Efficient Diagnostics and Therapy

**DOI:** 10.3389/fchem.2020.00561

**Published:** 2020-07-07

**Authors:** Andrew J. Gauger, Kian K. Hershberger, Lyudmila M. Bronstein

**Affiliations:** ^1^Department of Chemistry, Indiana University, Bloomington, IN, United States; ^2^Department of Physics, Faculty of Science, King Abdulaziz University, Jeddah, Saudi Arabia; ^3^A.N. Nesmeyanov Institute of Organoelement Compounds, Russian Academy of Sciences, Moscow, Russia

**Keywords:** theranostics, magnetic nanoparticles, polymer, engineering design, functionalization for cell targeting

## Abstract

Theranostics is a fast-growing field due to demands for new, efficient therapeutics which could be precisely delivered to the target site using multimodal imaging with enhancing auxiliary actions. In this review article we discuss theranostic nanoplatforms containing polymers and magnetic nanoparticles along with other components. Magnetic nanoparticles allow for both diagnostic and therapeutic (hyperthermia) capabilities, while polymers can be reservoirs for drugs and are easily functionalized for cell targeting. We focus on the most important design strategies to achieve optimal theranostic effects as well as the roles of different components included in theranostics, reviewing the literature from the last 5 years.

## Introduction

Over the years drug delivery systems have become so sophisticated that they are capable of combining therapeutic action from a drug or an external field with imaging capabilities such as magnetic resonance imaging (MRI), magnetic particle imaging (MPI), near infrared (NIR), and photoacoustic imaging (PAI), etc. The combination of imaging with therapy, called theranostics, allows for more precise delivery of a treatment, thus, increasing its efficacy. To fabricate theranostic drug delivery vehicles, one needs to combine in a single design the capacity to adsorb and release a drug, imaging properties, as well as some enhancing actions. We believe for the development of such efficient nanoplatforms, a combination of a polymer and magnetic nanoparticles (NPs), is favorable as the polymer can provide a reservoir for drugs and a platform for additional functionalization (for cell targeting or imaging), while magnetic NPs allow for MRI, MPI and hyperthermia—a cancer treatment which can be combined with a drug action. For the last 5 years, several review articles have been published discussing applications of hybrid nanomaterials based on bifunctional proteins, functional, and conducting polymers, dendrimers, biopolymers, etc. for diagnostics and therapies of serious diseases including cancer (Bonilla and Gonzalez, 2017; Deyev and Lebedenko, 2017; Kudr et al., 2017; Niu et al., 2017; Liu et al., 2018; Ray et al., 2018; Srinivasan et al., 2018; Aisida et al., 2019). Polymer-assisted magnetic NP assemblies showed a significant promise due to their controllable magnetic properties and collective functions (Li et al., 2020). Despite these excellent reviews, we believe there is a gap in understanding of key design elements needed to fulfill the desired theranostic functions for real life applications. In this review, we discuss the design of the most promising theranostics containing polymer and magnetic nanoparticles, allowing for the best combination of properties and possibilities of practical applications, analyzing literature from the last 5 years.

## Design Strategies

The major design strategies are 3-fold, focusing on (i) magnetic NPs with an enhanced magnetic response, (ii) a combination of magnetic and Au NPs, and (iii) sophisticated engineering solutions for fabrication of shells/containers.

### Magnetic NPs With Improved Magnetic Response

A typical way for increasing a magnetic response is iron oxide NP clustering which is discussed in section Nanoprecipitation of a Polymer and NPs. The other methods focus on varying a magnetic phase composition. A significantly improved magnetic response in terms of MRI and hyperthermia was achieved by combining soft magnetic phase (MnFe_2_O_4_ NPs) and hard magnetic phase (CoFe_2_O_4_ NPs) in bi-magnetic NP clusters (NPCs) stabilized by a biocompatible sodium dodecyl sulfate polymer (Vamvakidis et al., 2018). Paramagnetic ultrasmall NPs of Prussian blue [Fe^3+^(Fe^2+^(CN)_6_)] types containing Gd^3+^ ions in their exterior showed exceptional longitudinal relaxivities > 40 mM^−1^ s^−1^ per Gd^3+^ independently of the polymer shell (Fetiveau et al., 2019). In addition, a great photothermal effect and PAI of tumors *in vivo* were achieved.

### A Combination of Magnetic and Au NPs

Janus particles composed of trisoctahedral magnetite NPs and Au NPs coated with poly-L-lysine yielded a single nanoprobe with increased stability *in vivo* and efficient photothermal tumor ablation (due to gold) as well as enhanced MRI contrast properties due to the polymer layer (Abedin et al., 2018). Each constituent of these multicomponent particles plays an important role in theranostic applications. Other examples of a combination of gold and magnetic NPs were reported by Cherkasov et al. (2020), Wang et al. (2018), and (Sun et al., 2016).

### Engineering Design

**Figure 1:**
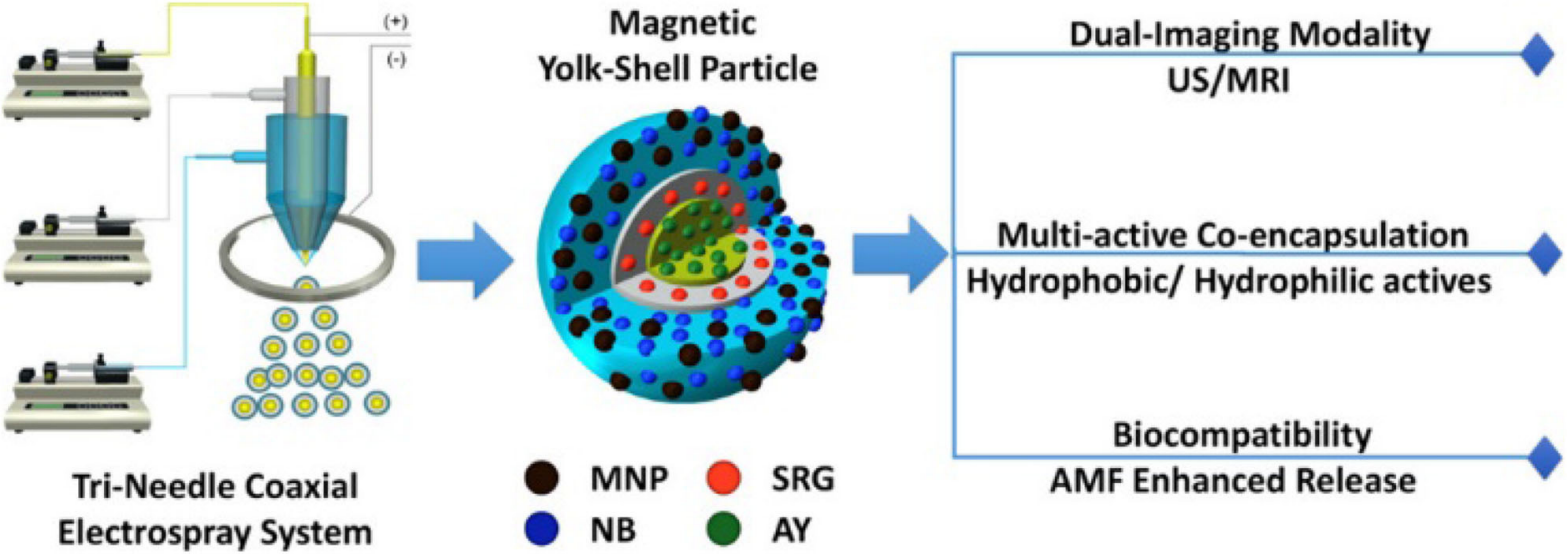
Schematic representation of tri-needle coaxial electrospray system for developing YSPs (Zhang C. et al., 2017). Reproduced with the permission of the copyright holder (American Chemical Society).

Zhang et al. developed sophisticated yolk-shell particles (YSPs) from simple components such as poly(ε-caprolactone) (PCL), silicone oil, and magnetite NPs (MNP) using tri-needle coaxial electrospray engineering to create a Fe_3_O_4_ NP-PCL shell surrounding an interfacing silicone oil layer, located around a PCL core (Figure 1; Zhang C. et al., 2017). The authors illustrated a possible simultaneous encapsulation and delivery of different drugs using the separate compartments of the YSPs. This situation was simulated using Nile blue (NB) and acridine yellow (AY)—model hydrophilic compounds—and Sudan red G (SRG) as a model for a hydrophobic drug. Dual-mode resonance (ultrasonic and magnetic) as well as multiple mechanisms of drug release (inversion, applied magnetic field, acoustic waves) and the possibility of using different polymers and NPs make these theranostics especially promising as universal systems for therapy and diagnostic.

High-power ultrasound was utilized to develop theranostics for gene silencing capabilities via a one-pot method (Israel et al., 2016). The NPs were functionalized with a polyethylene imine (b-PEI25) and a G2 PAMAM dendrimer biopolymer for attachment of small interfering RNA (siRNA) and decreasing cytotoxicity. This resulted in effective gene silencing (90–95%) combined with T2 MRI capabilities.

## Polymeric Component

### Coating of Magnetic NPs With a Polymer

An *in-situ* surface polymerization was developed to coat magnetite NPs with polypyrrole as well as poly(3,4-ethylenedioxythiophene):poly(4-styrenesulfonate; PEDOT:PSS; Yan et al., 2017). A conjugated polymer here allows for NIR absorbance. Powerful multimodal imaging capabilities of the NPs were utilized through PAI and MRI, while efficient hyperthermia resulted in effective tumor-ablation in mice.

Polyarabic acid, which is a major component of acacia gum, is biocompatible and facilitates penetration of cell membranes. Coating of magnetite NPs with polyarabic acid followed by further functionalization and DOX loading allowed fabrication of theranostic nanosystems with excellent cell penetration, DOX uptake, and pH sensitive DOX release in breast cancer tumor cells (Patitsa et al., 2017). Additionally, the NPs demonstrated great biocompatibility, minimal cytotoxicity, and contrasting properties comparable to current commercial agents. Coating with poly(ethylene glycol) (PEG), dextran, and chitosan allowed for better control over the polymer coat properties, enhancing both MRI and hyperthermia applications (Zahraei et al., 2016).

A novel nanotheranostic platform was developed from magnetite NPs conjugated to cyclodextrin polymer nanosponges, further functionalized with folic acid (FA) (Gholibegloo et al., 2019). The drug curcumin was then loaded into the lipophilic central cavity of cyclodextrins and a magnetic field was applied to guide the system toward the tumor whose acidic pH causes release of the drug.

A core-shell magneto-fluorescent nanogel was developed with iron oxide NP cores and a photoluminescent shell, where comacromers [PEG-maleic acid-glycine] were linked together by a crosslinker (Vijayan et al., 2019). The nanogel was found to possess high cytocompatibility, good cellular uptake, as well as fluorescent imaging and hyperthermia treatment capabilities. Crosslinking was utilized in another nanotheranostic platform to combine α-lactalbumin to itself via PEG and glutaraldehyde (Delavari et al., 2019). The resulting polymer was then attached to magnetite NPs using PEI. The redox sensitive protein complex holding DOX allowed for targeted release of the chemotherapeutic agent once in the acidic environment of a tumor.

In another example, a thermo-responsive fluorescent polymer (TFP) was conjugated to the surface of iron oxide magnetic NPs for use as a degradable and non-toxic theranostic agent (Pandey et al., 2020). For this, poly(N-isopropylacrylamide) (PNIPAM), allylamine and a fluorescent polymer were copolymerized and conjugated to NPs via free radical polymerization to form TFP-NPs. *In vitro* studies demonstrated low cytotoxicity and high biocompatibility of DOX loaded polymer-NP conjugates.

Yan et al. showed that the thickness of the PNIPAM coat around iron oxide NPs plays an important role in the drug uptake and MRI properties (Yan et al., 2017). It was established that thicker layers of PNIPAM translated to a higher uptake of DOX but subsequently led to a slower release and longer times to reach the target area during magnetic drug delivery.

### Nanoprecipitation of a Polymer and NPs

A pH switch nanoprecipitation method has been utilized for encapsulation of iron oxide NPs by a copolymer-drug conjugate including a tumor homing peptide (iRGD) with tumor specificity (Herranz-Blanco et al., 2016). The nanosystem showed increased lysosomal escape due to presence of poly(histidine) via the proton sponge effect and subsequent release of the polymer into the cytoplasm. The magnetic response was strong due to clustering, allowing for magnetic guided therapy. Additionally, intracellular cleavage of the DOX-polymer linkage was observed, allowing for efficient DOX delivery and accumulation in the nuclei of tumor cells.

Micelles prepared by nanoprecipitation of poly-(N-ε-carbobenzyloxy-L-lysine) grafted hyaluronan copolymer in the presence of iron oxide NPs and DOX were designed for targeted tumor diagnosis and treatment (Yang et al., 2020). The copolymer used in coating consisted of polysaccharides and polypeptides linked by disulfide bonds which, once reduced by the high concentration of glutathione (GSH) in the tumor tissue, released DOX. *In vitro* studies using HepG2 cells showed accelerated drug release over a 24 h period from these redox-sensitive theranostic NPs and increased intracellular uptake under a high GSH concentration, which mimics tumor cell conditions.

### Self-Assembly in the Formation of Smart Materials

**Figure 2:**
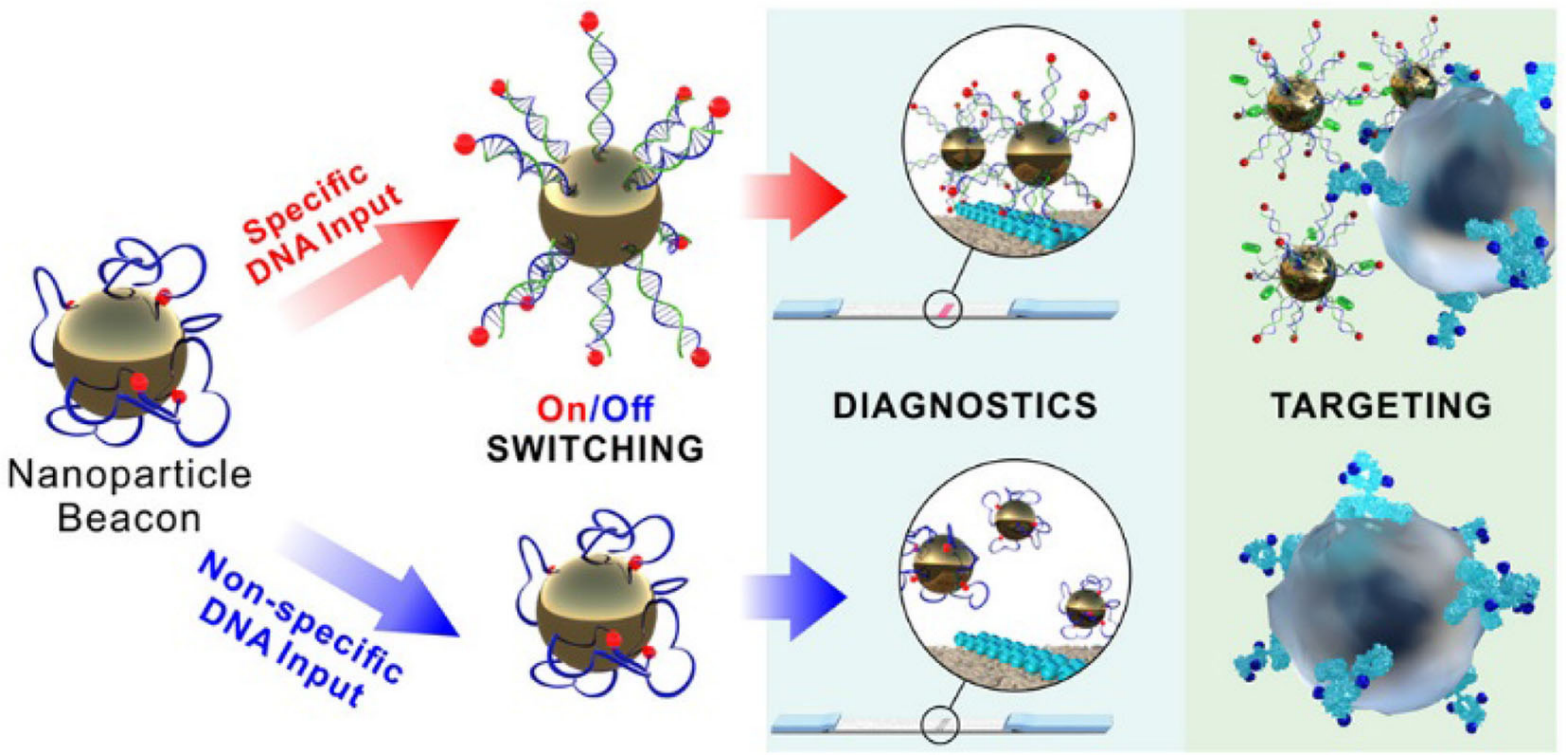
Schematic representation of the NP beacon action (Cherkasov et al., 2020). Reproduced with the permission of the copyright holder (American Chemical Society).

A NP beacon based on gold and ferrihydrite NPs coated with a flexible polymer (custom designed amino-group terminated oligonucleotides with terminal biotin) was fabricated by conjugation using streptavidin and DNA to form a highly sensitive input-switchable structure (Cherkasov et al., 2020). This structure controls accessibility of the terminal receptor for binding to the target (Figure 2). Here, DNA serves as a molecular cue to cause the smart material to undergo a serious of transformations allowing them to bind to cancer cells and initiate drug release. Biotin affinity to the gold surface shielded it from interaction until the specific input DNA turned the complex on so that biotin could interact with the target (streptavidin). These amazing constructs demonstrate an unusual signal transmittance between a biochemical cue (input) and a nanoprobe (targeting receptor) via surface polymer interactions and hold promise of remarkable future applications.

## Surface Modifications

### Fighting Phagocytosis With Self-Peptide

The mononuclear phagocyte system (MPS) poses as a barrier to NP delivery systems *in vivo*, and most delivery vehicles use a “passive stealth” approach through the presence of PEG or zwitterionic polymers, which delay the coating of nanovehicles with serum proteins and thus phagocytosis by the MPS. However, in the body, cell surface protein CD47 displayed on host cells interacts with phagocyte surface receptors, thus promoting anti-phagocytosis and acting as a “self-peptide” for the MPS to distinguish host from foreign particles. Zhang et al. explored an assembly of biodegradable poly(lactide-glycolide)-PEG (PLGA-PEG) with synthetic CD47 self-peptide for interactions with the SIRPα receptor expressed on phagocytes, i.e., an “active stealth” approach within the body (Zhang K.-L. et al., 2017). Also, iron oxide NPs absorbed anticancer drug through self-assembly into micelles, allowing for increased blood circulation, imaging, and drug delivery *in vivo*.

### Modification for Tumor Targeting

Functionalization of core-shell iron oxide NP-polymer constructs with FA allows for enhanced targeting and uptake in tumor cells via FA receptor mediated endocytosis (Huang et al., 2017; Roy et al., 2017; Gholibegloo et al., 2019). Functionalization with lysine and cysteine derivatives allowed for enhanced tumor cell ligand targeting and uptake (Wei et al., 2017). A tissue-type plasminogen activator peptide was employed for modification of a magnetic construct and targeting pancreatic cancer cells (Dobiasch et al., 2016).

### Use of Surfactant for Crossing Blood Brain Barrier

The blood brain barrier (BBB) often prevents crossing of theranostic agents into the brain, and thus poses a significant hurdle for any potential anticancer treatment targeting brain tumors. Iron oxide NPs modified with PEG, PEI, and Tween 80 (non-ionic surfactant) along with an applied magnetic field allowed for active penetration of the BBB *in vivo*, suggesting future potential for theranostic delivery through the BBB via Tween-NP conjugates (Huang et al., 2016). Other polymer based surfactants such as Brij-35, Pluronic F68, and Vitamin E-TPGS improved passage through BBB allowing for access to brain tumors such as glioblastoma (Luque-Michel et al., 2019).

It is noteworthy that lyophilization was efficiently used for preservation of theranostics with zero effect on physical, chemical, and magnetic properties after prolonged storage (Yang et al., 2017; Luque-Michel et al., 2019).

## Diagnostics

### MRI and MPI

Theranostic nanoplatforms with iron oxide NPs are normally utilized as T2 (negative) MRI contrast agents. Considering that MRI measures a proton response, NPs with higher magnetization and higher loading of water protons in a polymeric shell make a better MRI contrast agent (Liao et al., 2017; Yang et al., 2020). Polymer-NP constructs containing Gd^3+^ complexes (Roy et al., 2016; Fetiveau et al., 2019) or Fe^3+^- terpyridine complexes (Patra et al., 2018) can provide highly efficient T1 (positive) MRI contrast.

MPI—a comparatively recent imaging method—is based on a direct quantification of spatial distribution of iron oxide NPs in a tissue as a magnetization response to a magnetic field. It provides a higher contrast than that of MRI. A polymer-NP system showed promising MPI contrast properties (Rost et al., 2020).

### Fluorescence Imaging

Normally, fluorescence imaging is performed along with MRI by using a fluorescently labeled polymer (Pandey et al., 2020). For example, functionalization of iron oxide NPs with the two- photons fluorescent dye labeled polymer yielded a nanoprobe capable of quantifying pancreatic Beta cell mass (BCM), an effective indicator of onset of type 2 diabetes (Xin et al., 2020). Dye release was achieved via the acidic environment of the Beta pancreatic cells, thus allowing for fluorescence detection of Beta cells via confocal one-photon and two-photon microscopy. Furthermore, the nanoprobe displayed powerful inhibition of the toxic aggregation of human islet amyloid polypeptide, the suspected cause of Beta cell degeneration observed with type 2 diabetes. Magneto-fluorescent nanogels discussed in Section Nanoprecipitation of a Polymer and NPs displayed dual emissions (red and green) in Hela cells under different excitation wavelengths showing that this nanogel has fluorescence imaging applications toward cancer cells (Vijayan et al., 2019).

### Photoacoustic Imaging

PAI is a comparatively novel imaging modality, based on optical (laser) excitation and ultrasound imaging to detect sound waves (Attia et al., 2019). It is often combined with other imaging methods to impart multimodality and higher accuracy of imaging. Fetiveau et al. observed a strong photo-acoustic signal intratumorally after irradiation at ~808 nm of Gd-containing Fe^3+^[Fe^2+^(CN)_6_] NPs (Fetiveau et al., 2019). Lu et al. combined PAI, MRI and computer tomography utilizing dendrimer-stabilized nanoflowers containing Au and ultrasmall iron oxide NPs (Lu et al., 2018). Multimodality imaging including, PAI, MRI and fluorescence was demonstrated for fluorescent Janus nanostructures (Song et al., 2019).

## Therapeutic Properties

### Magnetic Delivery of Anti-cancer Agents

Multiblock polyurethanes (MPU) containing soft and hard polymer linkages formed iron oxide NPCs as a nanovehicle capable of magnetically targeted delivery *in vivo* (Wei et al., 2017). PEG connected by benzoic-imine linkage allowed for pH sensitive release of an anticancer drug in extracellular acidic environments. Overall, the MPU nanovehicle demonstrated powerful anticancer properties *in vivo* and MRI contrast properties.

Kumar et al. studied magnetic navigation steering capabilities of the nanostructures formed by maghemite NPs and thermoresponsive poly(2-ethyl-2oxazoline) in a synthetic blood vessel model (Kumar et al., 2018). High efficiency of magnetic steering navigation was achieved for all directions of blood flow studied, including angular, parallel, and antiparallel flow to the target destination combined with efficient release of anticancer drug (paclitaxel) at 41°C. Delavari et al. and Herranz-Blanco et al. also reported targeted delivery to tumor sites via magnetic guidance which allowed for increased DOX uptake in cancer cells (Herranz-Blanco et al., 2016; Delavari et al., 2019). This significantly improved cytotoxicity and apoptosis within the tumor while reducing those effects on healthy tissue.

### Reduction of Hypoxia

Hypoxia is a key aspect of tumor cell environments, contributing increased resistance to anticancer therapies. Loading of porous hollow Fe_3_O_4_ NPs with a FDA approved oxygen carrier, perfluorocarbon, was performed to study potential for decreasing hypoxia induced resistance to these treatments (Zhou et al., 2019). Modification of these NPs with lactobionic acid-containing block copolymer allowed for enhanced targeting of tumor cells and uptake via cleavage of the hydrophilic PEG segment by intratumoral GSH. Effective release of oxygen was achieved, leading to a decrease in hypoxia induced tumor resistance.

### Hyperthermia

Upon irradiation with an infrared laser or under the influence of an alternating magnetic field, many magnetic NPs undergo hyperthermia. It can be utilized in two major ways: enhanced drug release through the heat-expansion of a polymer coat (Zhang C. et al., 2017; Vijayan et al., 2019) and achieving temperatures above the minimum for inducing apoptosis (42°C) of surrounding cells (Thirunavukkarasu et al., 2018; Yar et al., 2018; Fetiveau et al., 2019) or by a combination of both methods (Vijayan et al., 2019). In all cases, once the nanomaterials had accumulated at the tumor site, they were triggered to reach required temperatures, resulting in a highly controlled and localized method for killing cancer cells.

### Gene Silencing

An enhanced interest has been shown for utilizing gene silencing and RNA interference techniques for cancer treatment. However, RNA interference is often limited due to fast degradation of siRNA by endonucleases prior to cell uptake. Functionalization of iron oxide NPs with chitosan and siRNA was performed for MRI contrast and RNA interference capabilities (Bruniaux et al., 2017). The addition of PEG and poly-L-arginine coating significantly increased the biocompatibility and siRNA transfection of the nanoplatform. Additionally, chitosan allowed for endosomal escape and thus pH sensitive release of siRNA into the cytoplasm. The use of polymer coated magnetic NPs as carriers for siRNA for gene silencing therapy was also reported by Israel et al. (2016).

## Summary and Outlook

What does the future hold in the field of magnetic theranostics? The literature shows that successful theranostics are based on three major principles: (i) multimodality of imaging allowing for high fidelity, (ii) efficacy of a therapeutic action often requiring additional functionalization for targeted delivery and an external field for an enhancement of the drug action, and (iii) potentials for robust scale up of the theranostic system production. The combination of all these factors can be realized in multicomponent systems, whose fabrication can be performed in a few steps. These requirements are met using tri-needle coaxial electrospray engineering resulting in YSPs. Moreover, this approach allows a wide variation of polymers, NPs, drugs, modifiers, etc., thus presenting the opportunity for a wide array of imaging modalities and therapeutic actions. Another promising fabrication strategy is self-assembly of multiple components into micelles (smart materials), where there is a molecular cue (for example, DNA), allowing one to trigger an ON-OFF mechanism of a therapeutic action. It is noteworthy that self-assembly is easy to scale up and to modify with various components.

At the same time, there is a number of shortcomings in this field, the major of which is a slow transition from laboratory studies to real world applications. This is often determined by high costs, fabrication issues, and the length of both preclinical and clinical studies. Thus, a concerted effort from scientists, engineers and clinicians is required to move the field forward.

## Author Contributions

All authors listed have made a substantial, direct and intellectual contribution to the work, and approved it for publication.

## Conflict of Interest

The authors declare that the research was conducted in the absence of any commercial or financial relationships that could be construed as a potential conflict of interest.
